# P2X2 Dominant Deafness Mutations Have No Negative Effect on Wild-Type Isoform: Implications for Functional Rescue and in Deafness Mechanism

**DOI:** 10.3389/fnmol.2017.00371

**Published:** 2017-11-13

**Authors:** Yan Zhu, Juline Beudez, Ning Yu, Thomas Grutter, Hong-Bo Zhao

**Affiliations:** ^1^Department of Otolaryngology, University of Kentucky Medical Center, Lexington, KY, United States; ^2^Centre National de la Recherche Scientifique, Unité Mixte de Recherche 7199, Laboratoire de Conception et Application de Molécules Bioactives, Équipe de Chimie et Neurobiologie Moléculaire, Strasbourg, France; ^3^Faculté de Pharmacie, Université de Strasbourg, Strasbourg, France; ^4^Department of Otolaryngology, Institute of Otolaryngology, Chinese PLA General Hospital, Beijing, China

**Keywords:** P2X2 receptor, mutation, deafness, dominant negative effect, functional restoration, ATP

## Abstract

The P2X2 receptor is an ATP-gated ion channel, assembled by three subunits. Recently, it has been found that heterozygous mutations of P2X2 V60L and G353R can cause autosomal dominant nonsyndromic hearing loss. However, the underlying mechanism remains unclear. The fact that heterozygous mutations cause deafness suggests that the mutations may have dominant-negative effect (DNE) on wild-type (WT) P2X2 isoforms and/or other partners leading to hearing loss. In this study, the effect of these dominant deafness P2X2 mutations on WT P2X2 was investigated. We found that sole transfection of both V60L and G353R deafness mutants could efficiently target to the plasma membrane, like WT P2X2, but exhibit a significantly reduced response to ATP stimulation. Both mutants reduced the channel conductance, but G353R mutation also altered the voltage dependency. Co-expression with WT P2X2 could restore the response to ATP. As the ratio of WT P2X2 vs. mutants increased, the response to ATP was also increased. Computer modeling confirmed that both V60L and G353R dominant-deafness mutant subunits do not have any negative effect on WT P2X2 subunit, when assembled as a heterotrimer. Improper docking or defective gating is the more likely mechanism for impaired channel function by these P2X2 deafness mutations. These results suggest that P2X2 dominant deafness mutations do not have negative effects on WT P2X2 isoforms, and that adding additional WT P2X2 could rescue the lost channel function caused by the deafness mutations. These P2X2 dominant deafness mutations may have negative-effects on other partners leading to hearing loss.

## Introduction

**Figure 1:**
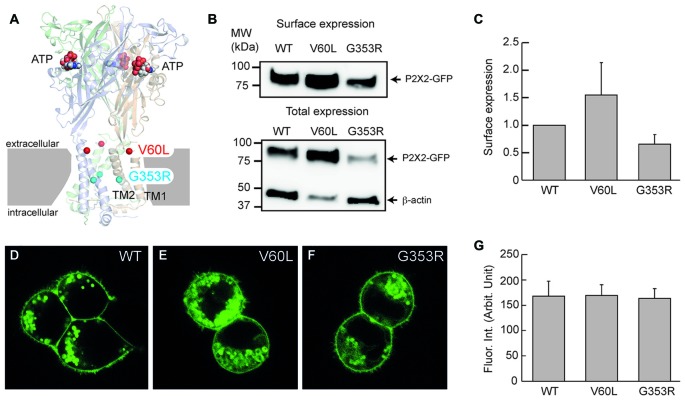
Membrane surface expression of P2X2 wild-type (WT), V60L and G353R. **(A)** Structure of the human P2X3 receptor solved in the presence of ATP (indicated by spheres; PDB ID: 5SVK; Mansoor et al., 2016). A functional P2X receptor is assembled by three subunits; each subunit is composed of two transmembrane domains (TM1 and TM2), an extracellular domain and two intracellular N- and C-termini. Locations of the corresponding human P2X2 deafness mutations V60L (red spheres) and G353R (cyan spheres) are indicated. **(B,C)** Cell surface expression of P2X2 WT-GFP, V60L-GFP and G353R-GFP is assessed by Western blot analyses of biotinylated receptors (data are mean ± SEM, *n* = 4 transfections). **(B)** shows representative Western blot analysis of cell-surface (top) and total expression (bottom) of WT and mutant receptors. Arrows indicate positions of P2X2-GFP and β-actin monomers. **(D–F)** Confocal images of HEK293 cells transfected with P2X2 WT-GFP, V60L-GFP and G353R-GFP. The surface expression on the plasma membrane is visible in both WT and mutants. Scale bar: 10 μm. **(G)** Quantitative analyses of the surface expression of the plasma membrane. Fluorescent intensity at the plasma membrane was measured. Data were expressed as mean ± SD.

ATP can act as an extracellular cell signaling molecule to influence cellular function in many aspects through the activation of purinergic (P2) receptors, which comprise ATP-gated ionotropic (P2X) and G protein-coupled metabotropic (P2Y) subgroups (Jacobson et al., 2002; North, 2002; Surprenant and North, 2009). A P2X receptor is a trimer, composed of three subunits (North, 2002; Kawate et al., 2009; Saul et al., 2013). Each subunit contains two transmembrane domains (TM), a large extracellular loop and intracellular N- and C-termini (Figure 1A). The extracellular domain contains three ATP-binding sites (Kawate et al., 2009; Hattori and Gouaux, 2012; Chataigneau et al., 2013). Upon ATP binding, motions of the extracellular domains induce opening of the channel transmembrane pore to allow K^+^ and Ca^2+^ influx.

ATP exists physiologically in the cochlear endolymph and perilymph (Muñoz et al., 1995), mainly released from gap junctional hemichannels (Zhao et al., 2005), in particular, Panx1 channels (Chen et al., 2015). It has been found that ATP in the cochlea can elevate intracellular Ca^2+^ concentration in hair cells to modify sound transduction and neurotransmission (Ashmore and Ohmori, 1990; Dulon et al., 1991; Sugasawa et al., 1996; Housley et al., 1999, 2013), mediate hearing sensitivity, extend the dynamic range of hearing (Housley et al., 1999; Thorne et al., 2004; Telang et al., 2010), synchronize auditory nerve activity during development (Tritsch et al., 2007; Tritsch and Bergles, 2010), and activate type II auditory nerves (Liu et al., 2015). In addition, ATP can activate P2X receptors to mediate stimulation of parasensory cation absorption (Lee et al., 2001). We also found that ATP can activate P2X receptors to mediate outer hair cell (OHC) electromotility (Zhao et al., 2005; Yu and Zhao, 2008), gap junctional coupling, K^+^-sinking and recycling, and endocochlear potential (EP) generation (Zhu and Zhao, 2010, 2012; Chen et al., 2015).

Recently, it has been found that heterozygous mutations of V60L (c. 178G > T) and G353R (c. 1057G > C) in P2X2 cause autosomal dominant nonsyndromic hearing loss DFNA41 (Yan et al., 2013; Faletra et al., 2014), further indicating that ATP-purinergic signaling has a critical role in hearing. However, the underlying deafness mechanism remains unclear. The fact that heterozygous mutations cause deafness suggests that the mutation may have a dominant-negative effect (DNE) on wild-type (WT) isoform and/or other partners. In this study, the effect of these dominant deafness P2X2 mutations on WT P2X2 was investigated. We found that both V60L and G353R dominant deafness mutants nearly lost all responses to ATP but had no DNE on WT P2X2. Based on their locations on the three-dimensional (3D) structure of the P2X receptor, deficiency of force-transferring from the ATP-binding site to the pore gating or defective gating is the more likely mechanism underlying impaired channel function.

## Materials and Methods

### P2X2 and Mutant Expression Plasmid Construction

Human P2X2-GFP plasmid and p.V60L-GFP plasmid were purchased from Origene (Rockville, MD, USA). The human P2X2-GFP plasmid was constructed with human P2X2 cDNA (NM_174873) cloned in pCMV6-AC-mGFP vectors. The mutant in human P2X2 V60L (c. 178G > T) cDNA was engineered using the Quikchange XLII site-directed mutagenesis kit (Stratagene, CA, USA) according to manufacturer’s instructions and cloned in pCMV6-AC-mGFP vectors. Human P2X2 G353R (c.1057G > C)-GFP plasmid was made in-house by the same materials and method. Presence of the desired mutations and absence of other non-specific mutations were verified by sequencing the entire gene.

### HEK 293 Cell Culture and P2X2 Transfection

HEK 293 cells were cultured in DMEM with 10% fetal bovine serum and 100 U/ml penicillin at 37°C in a 5% CO_2_ incubator. Cells were trypsinized and HEK 293 cells were seeded at a density of ~100,000 cells per well on a 24-well plate and incubated overnight. Then, the medium in the wells was replaced with fresh DMEM plus 10% FBS and cells were transfected with P2X2-GFP, V60L-GFP and G353R-GFP plasmids (10 μg) using Lipofectamine 2000 (Invitrogen) following manufacturer’s instructions. After 24–48 h, the successful transfectants were identified under the fluorescent microscope and captured by a Leica confocal microscope (Leica TCS SP2) equipped with 40× and 100× apochromatic oil objectives with a fixed set of laser and image collection parameters. All images were saved in the TIFF format.

### Quantitative Analysis of Cell Surface Membrane Expression

The confocal images were quantitatively analyzed by NIH image software (Bethesda, MD, USA). The fluorescent intensity at the plasma membrane was measured by the profile plotting function (Yu et al., 2006, 2008). For each cell, the place of non-apparent cytoplasm-labeling was selected. A line perpendicular to the cell surface was drawn cross the cell surface and the intensities of pixels along the line were recorded by the profile plotting. The mean of fluorescence intensity at the plasma membrane was measured by Gaussian fitting [*y* = *a* * *log*(−((*x* − *x*_0_)/*b*)^2^)]. Then, the measured intensities were averaged.

Cell surface expression of P2X2, V60L and G353R was also verified by biotinylation-Western blotting assay. As previously described (Jiang et al., 2010), a membrane-impermeant, thiol-cleavable amino-reactive reagent, sulfosuccinimidyl-2-(biotinamido)ethyl-1,3′-dithiopropionate (EZ-Link™ Sulfo-NHS-SS-Biotin, ThermoFisher Scientific, France), was used. P2X2 WT or mutants (10 μg) were transfected into HEK-293 cells using calcium phosphate precipitation. After 24 h, cells were solubilized in lysis buffer and the supernatant was incubated with neutravidin-agarose beads (ThermoFisher Scientific, France) overnight. After DTT cleavage, protein samples were run on a 4%–15% SDS-PAGE in Tris/Glycine/SDS running buffer (Bio-Rad, France). For total expression, 15 μl out of 200 μl of solubilized cells were loaded on the SDS-PAGE. After electrophoresis, proteins were transferred to a nitro-cellulose membrane. This membrane was blocked for 30 min with TPBS (PBS supplemented with 1% non-fat dry milk, 0.5% bovine serum albumin, and 0.05% Tween 20) and incubated in the same buffer overnight at 4°C with both anti-mGFP antibody, diluted at 1:500 (OriGene, Germany) and anti-β-actin antibody (Sigma-Aldrich, France), diluted at 1:5000. Then, the membrane was incubated with peroxidase-conjugated sheep anti-mouse antibody for 2 h at room temperature, diluted at 1:10,000 (GE Healthcare Life Sciences, France). Blots were developed with the Amersham ECL Prime Western blotting detection reagent (Dominique Dutscher, France). The image was captured with Amersham Imager 600 and the intensity of the band corresponding to surface expression was measured (Figure 1B). The measured intensities were normalized to the intensity of P2X2 WT and averaged (Figure 1C).

### Patch-Clamp Recording

Patch clamp recording was performed as we previously reported (Zhu and Zhao, 2010, 2012). The culture cells were rinsed with normal extracellular solution (NES; 130 NaCl, 5.37 KCl, 1.47 MgCl_2_, 2 CaCl_2_, 25 Dextrose and 10 HEPES in mM; 300 mOsm and pH 7.2) three times. Then, the culture cells were continually perfused with NES. A single, isolated cell with strong fluorescence was selected and classical whole-cell recording was performed using Axopatch 200B (Molecular Devices, CA, USA). Patch pipettes were filled with a normal intracellular solution that contained (in mM): 140 KCl, 5 EGTA, 2 MgCl_2_ and 10 HEPES, pH 7.2 with initial resistance of 2.5–3.5 MΩ in the bath solution. Data collection was performed with jClamp (SciSoft, New Haven, CT, USA). The signal was filtered by a 4-pole low-pass Bessel filter with a cut-off frequency of 2 kHz and digitized utilizing a Digidata 1322A A/D-D/A board (Molecular Devices, CA, USA). The patch clamp recording was conducted at room temperature (23°C).

The cell was held at −80 mV. The holding current, cell capacitance, and other recording parameters were continuously recorded in jClamp as we previously reported (Zhu and Zhao, 2010, 2012). The current-trace for ATP stimulation was recorded when the cell was held at −80 mV. In some cases, the current-voltage (I-V) curve was measured at the max point in the trace of ATP-evoked inward current by voltage-steps from −150 mV to 70 mV for 100 ms in 10 mV increments. The I-V curve was plotted by average values of the steady-state currents in last 20 ms of the voltage step stimulation. The conductance was calculated by the current divided by the membrane potential (*V*_m_), which was corrected for pipette series resistance (*R*_s_).

### Chemicals and Chemical Application

All chemicals were purchased from Sigma-Aldrich (St. Louis). ATP was applied by a Y-tube or a bath perfusion system (Yu and Zhao, 2008; Zhu and Zhao, 2010, 2012).

### Data Collection, Analysis and Display

All data were collected from at least three different experiments. Data were plotted by SigmaPlot and statistically analyzed by SPSS v18.0 (SPSS Inc. Chicago, IL, USA). Data were expressed as mean ± SEM other than indicated in text.

## Results

### Plasma Membrane Targeting of P2X2 Deafness Mutations

The P2X2 receptor is a membrane protein and functions on the cell surface. Figure 1 shows that even though P2X2 V60L and G353R mutations had expression in the cytoplasm, they could target to the plasma membrane and had good expression on the cell surface similar to WT P2X2 (Figures 1D–G). Quantitative analyses show that the measured fluorescent intensities of P2X2 WT, V60L and G353R at the plasma membrane were 167.8 ± 29.3 (*n* = 12), 168.0 ± 22.3 (*n* = 19), and 163.3 ± 19.3 (*n* = 13), respectively. There was no significant difference among P2X2 WT, V60L and G353R membrane expression (Figure 1G, *p* = 0.41, one-way analysis of variance (ANOVA)). Using cell-surface biotinylation Western blotting assay, we further confirmed the cell-surface expression of V60L and G353R mutants (Figures 1B,C), and that there was no significant difference in the level of surface expression among V60L, G353R and WT P2X2 (Figure 1C, *p* = 0.16, one-way ANOVA).

### Absence of ATP Responses in P2X2 Mutants

**Figure 2:**
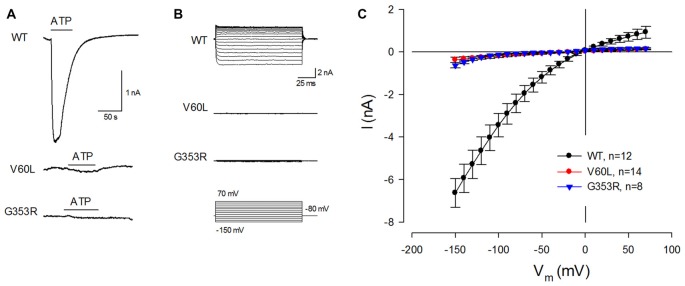
P2X2 deafness mutations eliminate current responses to ATP stimulation. **(A)** An ATP-evoked inward current is visible in a P2X2 WT-transfected cell but eliminated in mutant-transfected cells. Horizontal lines represent ATP (36 μM) perfusion. Cells were held at −80 mV. **(B)** ATP-evoked current traces across the applied voltage range from −150 mV to 70 mV in P2X2 WT and mutant transfected cells. Little current responses are visible in V60L and G353R transfected cells. **(C)** The current-voltage (I-V) relationships were plotted by average values of the steady-state currents in last 20 ms of 100 ms voltage stimulations.

These deafness mutants, however, lost responses to ATP stimulation. Figure 2 shows that application of 36 μM ATP could evoke a large inward current in WT P2X2 transfected cells. However, there was almost no ATP-evoked inward current in V60L or G353R transfected cells (Figures 2A,B). At −80 mV, the ATP-evoked inward currents in P2X2 WT, V60L and G353R transfected cells were −2.42 ± 0.43 (*n* = 12), −0.15 ± 0.05 (*n* = 14), and −0.07 ± 0.01 (*n* = 8) nA, respectively (Figure 2C). In comparison with that in WT P2X2, the ATP-evoked inward currents in V60L or G353R transfected cells were significantly reduced (*p* < 0.001, one-way ANOVA with a Bonferroni correction). At high ATP concentration (1 mM), the current responses of WT P2X2 at −80 mV were −7.07 ± 1.48 nA (*n* = 4). However, apparent responses to ATP stimulation were still significantly reduced in the mutants V60L and G353R in comparison with WT P2X2. The recorded currents of V60L and G353R mutants at −80 mV were −0.18 ± 0.07 (*n* = 4), and −0.11 ± 0.03 (*n* = 3) nA, respectively (*p* < 0.001, one-way ANOVA with a Bonferroni correction).

### Responses of ATP in Co-Transfection of Mutants with WT P2X2

**Figure 3:**
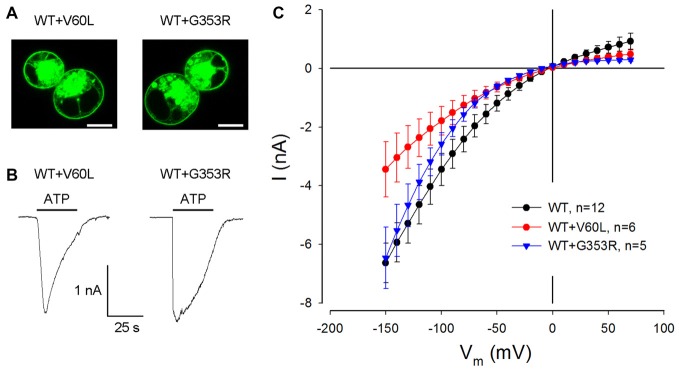
Restoration of ATP-evoked current responses in P2X2 WT and mutant co-transfected cells. **(A)** Membrane surface expression is visible in the co-transfection of V60L or G353R with P2X2 WT fused with GFP. Scale bar: 10 μm. **(B)** The ATP-evoked inward currents in co-transfection of WT P2X2 with V60L or G353R mutations. The horizontal line bars represent perfusion of 1 mM ATP. The cells were held at −80 mV. **(C)** The I-V relationship of ATP-evoked responses in co-transfection of V60L or G353R mutant with WT P2X2. The ATP-evoked inward currents are reduced but not completely abolished in WT and mutant co-transfected cells.

We further tested whether these dominant deafness mutations have a negative effect on WT P2X2. Figure 3 shows the membrane expression and ATP responses in co-transfection of WT P2X2 and deafness mutations. As shown in V60L and G363R sole-transfected cells (Figures 1E,F), good surface expression was visible in co-transfection of WT P2X2 and these mutants (Figure 3A). Also, there were apparent ATP-evoked inward currents in these co-transfected cells (Figure 3B). With a 1:1 co-transfection ratio, ATP-evoked current in P2X2 WT and V60L co-transfected cells was half the value of the current recorded from P2X2 WT sole-transfected cells (Figure 3B). In co-transfection of P2X2 WT and G353R, the ATP-evoked inward current was even larger. At 36 μM ATP stimulation, the evoked currents in P2X2 WT, WT+V60L and WT+G353R transfected cells at −80 mV were −2.42 ± 0.43 nA (*n* = 12), −1.26 ± 0.34 nA (*n* = 6), and −1.58 ± 0.23 nA (*n* = 5), respectively. As the cell was hyperpolarized, the ATP-evoked currents in co-transfection became large. At −150 mV, the ATP-evoked inward currents in P2X2 WT, WT+V60L and WT+G353R transfected cells were −6.63 ± 0.67 nA (*n* = 12), −3.44 ± 0.94 nA (*n* = 6), and −6.46 ± 1.04 nA (*n* = 5), respectively. The ATP-evoked inward current in WT+V60L co-transfected cells retained half the value of the current recorded from P2X2 WT transfected cells. However, the ATP-evoked inward current in WT+G353R co-transfected cells became as large as the current recorded from P2X2 WT transfected cells (Figure 3B).

**Figure 4:**
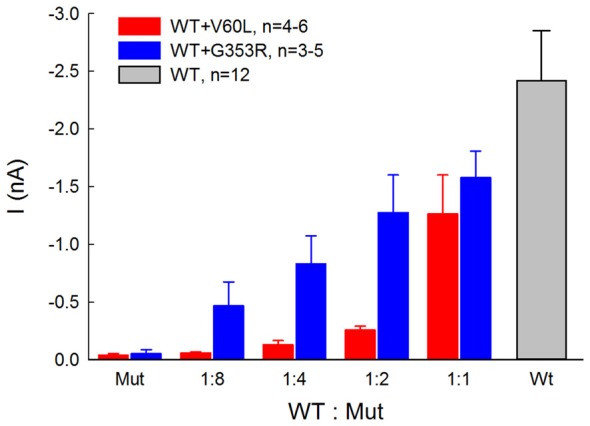
ATP-evoked responses in the co-transfection of WT and P2X2 deafness mutation V60L and G353R are ratio-dependent. The responses were recorded at ATP = 36 μM and V_h_ = −80 mV. In comparison with the responses of sole-transfection of mutants, ATP responses in the co-transfection dramatically increased as the co-transfection ratio was increased.

Figure 4 shows that the ATP-evoked responses in co-transfection of WT and deafness mutations were dependent upon the co-transfection ratio of WT vs. mutant. In comparison with sole deafness mutant, the responses to ATP in the co-transfection dramatically increased. Also, as the co-transfection ratio of WT vs. mutant was increased (i.e., WT P2X2 was increased), the responses were increased, demonstrating a ratio-dependent manner.

### Gating and Voltage Dependence of Deafness Mutations

**Figure 5:**
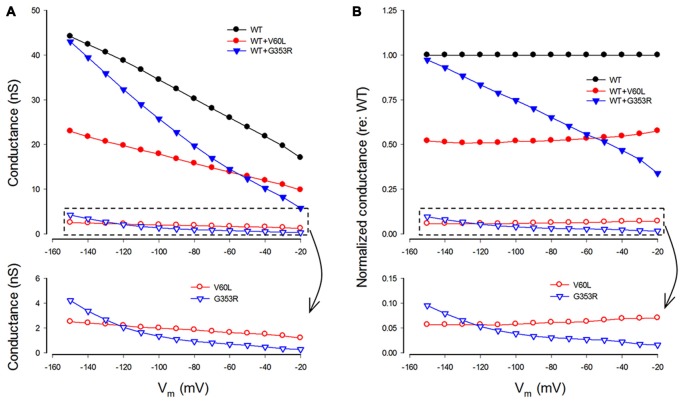
Conductance and voltage dependence of P2X2 WT and deafness mutants. **(A)** Conductance of P2X2 WT and co-transfection with V60L and G353R mutations. The lower graphic is the amplified plot of the conductance of the cells transfected with mutants alone. The conductance is calculated from current responses evoked by ATP stimulations. **(B)** Normalized conductance to P2X2 WT conductance. The lower graphic is the amplified plot for transfected with mutants alone.

We also analyzed conductance and voltage dependence of P2X2 V60L and G353R mutations (Figure 5). Under 36 μM ATP stimulation, the conductance of WT P2X2, V60L and G353R at −80 mV was 30.2 ± 5.43 nS (*n* = 12), 1.85 ± 0.57 nS (*n* = 14), and 0.93 ± 0.15 nS (*n* = 8), respectively (Figure 5A). The conductance of V60L and G353R was significantly reduced (*p* < 0.001, one-way ANOVA with a Bonferroni correction). As cells were depolarized, the conductance of both P2X2 WT and V60L was reduced. However, the decrements in WT P2X2 and V60L were almost parallel to each other, even though the conductance of V60L mutation was dramatically reduced (Figure 5A). The normalized conductance of V60L to the WT conductance appeared flat and parallel to that in WT P2X2 in the stimulus voltage range (Figure 5B, bottom), indicating that mutation of V60L has similar voltage dependence to WT P2X2. However, unlike V60L mutation, G353R mutation not only reduced conductance but also altered the voltage dependence (Figure 5). The normalized conductance of G353R was not parallel to WT P2X2 and was reduced as cells were depolarized (Figure 5B, bottom), indicating that G353R mutation not only decreases channel conductance but also reduces the sensitivity to voltage.

In the co-expression of P2X2 WT and mutations, the conductance became larger than that of the sole-expression of either mutant. With a 1:1 co-transfection ratio, the conductance of P2X2 WT and V60L co-transfection at −80 and −150 mV was 15.8 ± 4.22 and 22.9 ± 6.29 nS (*n* = 6), respectively, at 36 μM ATP stimulation. They also had a conductance half of that of WT P2X2 (Figures 5A,B). The voltage dependence of co-transfection of P2X2 WT and V60L was also similar to that of WT P2X2; the normalized conductance of WT and V60L co-transfection was parallel to WT P2X2 as well (Figure 5B). In the co-transfection of G353R and WT P2X2, the conductance was also larger than that in sole G353R transfection (Figure 5). However, unlike co-transfection of P2X2 WT and V60L, the conductance in the co-transfection of P2X2 WT and G353R was increased as the cell was hyperpolarized. At −150 mV, the conductance was almost as large as WT P2X2. The normalized conductance of the co-transfection was also not parallel to WT P2X2 (Figure 5B).

### Modeling Analysis of the Effect of Dominant Deafness Mutations on WT P2X2

**Figure 6:**
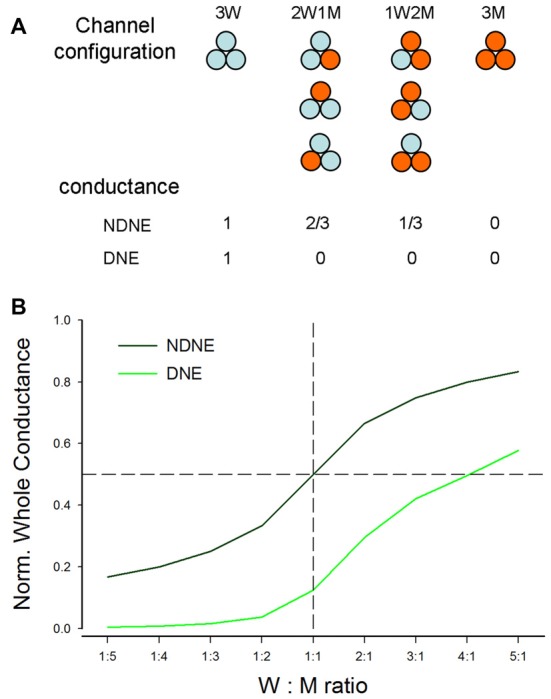
Modeling analysis of the dominant negative effect (DNE) and no dominant negative effect (NDNE) in co-expression of P2X2 WT and mutation. **(A)** Channel configuration and corresponding conductance in the co-expression with DNE and NDNE. **(B)** The predicted conductance by modeling with DNE and NDENE in co-transfection with different expression ratios of WT vs. mutant.

**Figure 7:**
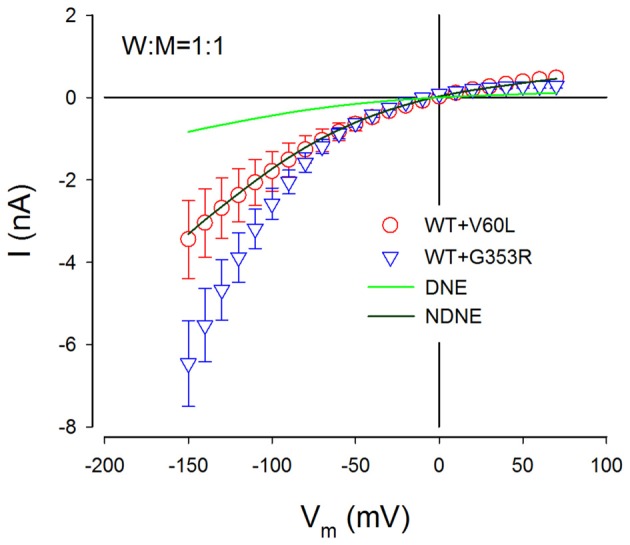
NDNE of P2X2 dominant deafness mutations on WT P2X2. Solid lines represent the current responses predicted from modeling with DNE and NDNE at the ratio of W:M = 1:1. Red and blue empty symbols represent ATP-evoked currents from co-transfection of P2X2 WT with V60L and G353R, respectively, which are adapted from Figure 3B. The current responses from the co-transfection of V60L with WT P2X2 perfectly match the model prediction with NDNE. The currents of co-transfection of P2X2 WT and G353R are also larger than the model prediction, indicating that there is no negative effect on WT P2X2 function.

We further used computer modeling to assess the effect of dominant deafness mutation on WT P2X2 (Figures 6, 7). A P2X2 channel is a trimer and is assembled by three subunits (Saul et al., 2013). In co-transfection, the WT (W) and mutant (M) isoforms can form four types of channel configurations, WWW, WWM, WMM, and MMM, for a trimeric channel. The probability of each channel configuration can be described by the binomial distribution (Colquhoun and Hawkes, 1981; Uteshev, 1993):

$$P(k,p)=C(n,k)*p^{k}*{\left(1-p\right)}^{n-k}$$

where, *P* is the probability that a channel has *k* WT subunits, *p* is the probability of the WT channel from the pool of available subunits (*n*), and (1-*p*) is corresponding probability of the mutant channel. In the co-expression of WT and mutant, *n* is determined by the ratio of WT vs. mutant. For example, *n* is 2 for the ratio of WT:Mut = 1:1, 3 for the ratio of WT:Mut = 1:2, and so on.

If the mutant has no negative effect on WT isoforms and each subunit is functionally independent (Figure 6A), the conductance of WWW, WWM, WMM and MMM channels will be 1, 2/3, 1/3 and 0, respectively. However, if the mutant has a negative effect on WT isoforms, with the exception of WWW homomeric channels, all channels with a mutant isoform will lose function (Figure 6A). Figure 6B shows the conductance predicted by modeling at different co-transfection ratios with or without DNE. The conductance with the negative effect is much lower than that without the negative effect. For example, at the co-expression ratio of WT:Mut = 1:1 (indicated by a dashed vertical line in Figure 6B), the conductance without the negative effect is 0.5, whereas the conductance with the negative effect is 0.125 (Figure 6B).

Figure 7 shows the comparison of predicted current responses by modeling with or without the DNE with currents recorded from P2X2 WT with co-transfection of mutant of V60L and G353R. As shown by a dark green line in Figure 7, the prediction by the modeling without the DNE perfectly matches the current responses recorded from the P2X2 WT and V60L co-transfection (red empty circles in Figure 7), while the prediction by the modeling with the DNE (as shown by a green line in Figure 7) is much smaller than the recorded currents.

This simple model does not include changes in voltage dependence. The currents recorded from the co-transfection of G353R and WT P2X2 did not perfectly match the prediction by this model (blue empty triangles in Figure 7), due to G353R mutation also altered the voltage dependence (Figure 5). However, the currents recorded from the co-transfection of P2X2 WT and G353R were larger than the modeling prediction with the DNE (Figure 7), indicating that G353R mutation has no DNE on WT P2X2, too.

## Discussion

In this study, we found that both P2X2 deafness mutations V60L and G353R retained good surface expression on the plasma membrane but nearly lost all responses to ATP (Figures 1, 2). However, unlike V60L, G353R mutation also altered voltage dependence, decreasing the sensitivity to voltage as cells are depolarizing (Figure 5). Co-expression with WT P2X2 could partially restore the impaired function caused by the mutations (Figures 3, 4). Computer modeling analyses reveal that both dominant deafness mutations have no negative effect on WT P2X2 (Figures 6, 7). However, they may have different underlying channel-gating mechanisms for the loss of function.

As shown in Figure 1A, mutations V60L and G353R are located at different positions on the 3D structure. Mutation V60L localizes at the extracellular part of TM1, whereas mutation G353R localizes at the TM2 pore site. Recently, crystal structures of the zebrafish P2X4 (zfP2X4) and human P2X3 (hP2X3) receptors solved in apo and ATP-bound states revealed molecular motion of the extracellular domain following agonist binding (Kawate et al., 2009; Hattori and Gouaux, 2012; Mansoor et al., 2016). ATP-binding causes motion in the extracellular domains, which induces opening of the channel pore at the transmembrane domain. From the hP2X3 X-ray structure, residue V42 (equivalent to V60 in human P2X2) is located at last turn of the TM1, opposite to the hydrophobic I319 residue from another subunit (Mansoor et al., 2016). These two residues interact with each other in the resting, closed state (Jiang et al., 2003). Mutation V60L may form strong interactions with I333 (equivalent to I319 in hP2X3), which lock these residues in place and prevent channel gating. This will also impair the force transfer from the ATP-binding site at the extracellular domain to the channel pore at the transmembrane domain to open the channel. However, the mutation does not directly impair the function of the channel pore. Indeed, mutation of V60L only reduced the channel conductance but conferred similar voltage dependence to WT P2X2 receptors (Figure 5), supporting this concept.

Mutation G353R, however, not only reduced the channel conductance but also altered voltage dependence (Figure 5). The mutation of G353R localizes at the cytoplasmic vestibule of the gate (Kawate et al., 2009; Caseley et al., 2014). A recent study demonstrated that the flexibility of the residue of rat G342 (equivalent to G353 in the human P2X2 receptor) is important for gating (Habermacher et al., 2016). It has been reported that the substitutions of G342C and G342K in the rat P2X2 receptor can impair channel gating resulting in small or absence of ATP-evoked currents (Cao et al., 2009). Our results are consistent with these previous reports. The mutation of G353R in the human P2X2 receptor may share the same mechanism by impairing channel gating which leads to the sharp decrease of the ATP response.

Our data and computer modeling analysis also provide important information about P2X2 channel activity. The P2X2 receptor is a trimer, assembled by three subunits. However, the action of each subunit in the trimer is less understood. Our data show that docking with WT P2X2 can compensate the defective effect of mutants to restore the channel function, which matched the prediction by binomial modeling (Figures 6, 7). This suggests that each subunit in the P2X2 trimer can be individually functional. This is also consistent with previous reports that T336C mutant did not act in a dominant fashion (Stoop et al., 1999). It has been reported that subunits in P2X receptors also have positive cooperativity (Ding and Sachs, 1999, 2002). Indeed, we found that unlike V60L, G353R mutation in co-transfection with WT P2X2 demonstrated a nonlinear voltage-dependent current response (Figures 3, 5), larger than the prediction by a simple binomial model (Figures 6, 7). In particular, small portion of WT co-expressed with G353R mutation could dramatically increase the response to ATP (Figure 4). This may result from the positively cooperative effect on gating activity among subunits.

In the experiment, we found that co-transfection of deafness mutations with WT P2X2 could restore the mutation-induced deficiency in the response to ATP (Figures 3, 4). This may also provide important information for developing therapeutic strategies targeting this hearing loss. However, our present results showed that both V60L and G353R co-expressed with WT P2X2 could restore the lost channel function (Figures 3, 4), inconsistent with a recent report that co-transfection of P2X2 WT and V60L mutation has no response to ATP stimulation (Mittal et al., 2016). In that study, they also claimed that these P2X2 mutations could affect hydrolysis of ATP to influence P2X2 channel activity and function (Mittal et al., 2016), which is unreasonable. First, it is well-known that ATP-induced opening of P2X receptors is due to a simple binding process and does not require the hydrolysis of ATP (Kawate et al., 2009; Hattori and Gouaux, 2012; Jiang et al., 2012). Second, opening of P2X channels by ATP is very fast within milliseconds following the binding of ATP (Figures 2, 3), whereas the reported hydrolysis is very slow, happening in minutes to hours (Mittal et al., 2016). Finally, there was no evidence that there are no other proteins, especially ATPases, mixed in their ATP hydrolysis experiment to hydrolyze ATP. In the present study, we further showed that the restoration of the lost function by co-expression of V60L or G353R with WT P2X2 is dose-dependent; the response to ATP was increased as the ratio of co-expression with WT P2X2 was increased (Figure 4). This further suggests that co-expression with WT P2X2 could restore the lost channel function by not only G353R but also V60L mutation.

P2X2 receptor mutations induced DFNA41 is autosomal dominant deafness, which is caused by heterozygous mutants (Yan et al., 2013; Faletra et al., 2014). This suggests that the mutations may have the negative effect on WT P2X2 and/or other partner(s), thereby leading to hearing loss. However, we found that WT P2X2 co-transfected with V60L and G353R dominant-deafness mutations still retained robust responses to ATP stimulation (Figures 3, 4), i.e., dominant-deafness mutations did not abolish WT P2X2 function. Computer modeling analyses (Figures 6, 7) also confirmed that the mutations have no negative effect on WT P2X2. Furthermore, it has been found that P2X2-null mice have no hearing loss and demonstrate normal hearing (Housley et al., 2013). Taken together, these data suggest that P2X2 dominant deafness mutations V60L and G353R have no DNEs on WT P2X2, and that hearing loss caused by these P2X2 dominant-deafness mutations may be unlikely to result from sole loss of P2X2 receptor function. Other mechanisms, such as negative effect on other partners, may play a critical role in hearing loss. It has been reported that P2X receptors can cooperate with Panx1 channels to play an important role in ATP release (Khakh and North, 2012). Recently, we found that Panx1 knockout mice have hearing loss (Chen et al., 2015; Zhao et al., 2015), resulting from the reduction of ATP release in the cochlea and cochlear EP generation, thereby reducing auditory receptor current/potential (Chen et al., 2015). Panx1 mutation also caused hearing loss in humans (Shao et al., 2016). The P2X2 dominant deafness mutations may have a negative effect on Panx1 leading to hearing loss. This hypothesized mechanism needs to be further studied in the future.

## Author Contributions

H-BZ conceived the general framework of this study. YZ, JB, NY, TG and H-BZ performed the experiments and analyzed data. H-BZ and TG wrote the article. All authors reviewed the manuscript and provided the input.

## Conflict of Interest Statement

The authors declare that the research was conducted in the absence of any commercial or financial relationships that could be construed as a potential conflict of interest.
